# Immune checkpoint inhibitor–associated myocarditis: Case reports and a review of the literature

**DOI:** 10.1007/s12471-021-01655-7

**Published:** 2022-01-21

**Authors:** T. E. Osinga, S. F. Oosting, P. van der Meer, R. A. de Boer, B. C. Kuenen, A. Rutgers, L. Bergmann, T. H. Oude Munnink, M. Jalving, M. van Kruchten

**Affiliations:** 1 Department of Medical Oncology, University of Groningen, University Medical Centre Groningen, Groningen, The Netherlands; 2 Department of Cardiology, University of Groningen, University Medical Centre Groningen, Groningen, The Netherlands; 3 Department of Internal Medicine, Martini Hospital, Groningen, The Netherlands; 4 Department of Rheumatology and Clinical Immunology, University of Groningen, University Medical Centre Groningen, Groningen, The Netherlands; 5 Medical Clinic II, J.W. Goethe University, Frankfurt, Germany; 6 Department of Clinical Pharmacy and Pharmacology, University of Groningen, University Medical Centre Groningen, Groningen, The Netherlands

**Keywords:** Cardiac biomarkers, Cardio-oncology, Immune checkpoint inhibitor, Associated myocarditis, ICI-associated myocarditis

## Abstract

Immune checkpoint inhibitors (ICIs) are increasingly recognised to effectuate long-lasting therapeutic responses in solid tumours. However, ICI therapy can also result in various immune-related adverse events, such as ICI-associated myocarditis, a rare but serious complication. The clinical spectrum is wide and includes asymptomatic patients and patients with fulminant heart failure, making it challenging to diagnose this condition. Furthermore, the optimal diagnostic algorithm and treatment of ICI-associated myocarditis is unknown. In this review, we describe two cases on both ends of the spectrum and discuss the challenges in recognising, diagnosing and treating ICI-associated myocarditis.

## Introduction

Immune checkpoint inhibitors (ICIs) have led to a revolution in the treatment of a variety of cancers. Augmentation of immune responses by ICIs is key to inducing the desired anti-tumour effect. ICIs amplify the host T cell response against tumour antigens by targeting specific inhibitory signals in the T cell regulatory pathways, such as cytotoxic T lymphocyte–associated antigen 4 (CTLA-4), programmed cell death receptor 1 (PD-1) and programmed cell death ligand 1 (PD-L1).

However, ICI therapy is associated with a wide range of immune-related toxicities. Among these, ICI-associated myocarditis is a rare but life-threatening complication [1]. Little is known about the optimal algorithm to screen for and diagnose ICI-associated myocarditis. In this article, we describe two distinct cases: the first about a fulminant disease course and the second about a patient who was diagnosed by detection of asymptomatic elevated levels of troponin. After the case descriptions, we provide a review of the literature and discuss the challenges in recognising, diagnosing and treating ICI-associated myocarditis.

## Case 1

A 58-year-old man with a history of hypertension and diabetes mellitus was diagnosed with poor-risk metastatic clear cell renal cell carcinoma. Immunotherapy with ipilimumab (human monoclonal anti–CTLA‑4 antibody) 1 mg/kg and nivolumab (human monoclonal anti–PD‑1 antibody) 3 mg/kg intravenously, every 3 weeks for four cycles, was initiated [2–4]. On day 2 of the second cycle, the patient collapsed and was subsequently brought to the emergency department.

**Fig. 1:**
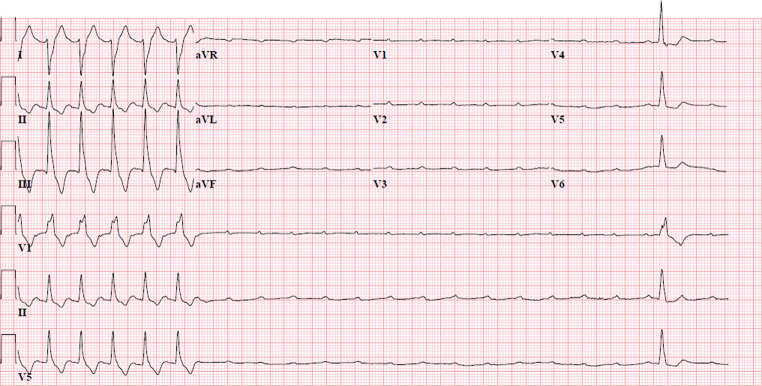
Electrocardiogram showing a third-degree atrioventricular block, initially without escape rhythm, but after several seconds an escape beat is visible

On physical examination, he was conscious and haemodynamically stable. The electrocardiogram (ECG) showed a third-degree atrioventricular (AV) block and ventricular escape rhythm (Fig. 1). Troponin T, creatine kinase (CK) and CK myocardial band (CK-MB) levels were 721 ng/l (normal < 14 ng/l), 459 U/l (normal < 117 U/l) and 34 U/l (normal < 25 U/l), respectively. At that time, baseline troponin values were not routinely measured in our hospital. The patient was admitted to the coronary care unit (CCU). Given the new-onset third-degree AV block, elevated cardiac markers and recent initiation of immunotherapy, ICI-associated myocarditis was suspected. Myocardial ischaemia, pulmonary embolism and viral myocarditis were considered less likely.

Viral panel testing was negative for Epstein-Barr virus, parvovirus B19, human herpes virus 6, enterovirus, hepatitis B virus and hepatitis C virus. Bedside echocardiography showed a left ventricular ejection fraction (LVEF) of 45–50% with signs suggestive of diastolic dysfunction. Despite intravenous isoprenaline therapy, episodes of bradycardia persisted, and a temporary external pacemaker wire was inserted. Prednisolone was started to treat ICI-associated myocarditis at a dose of 2 mg/kg/day intravenously, according to the existing literature [5].

**Fig. 2:**
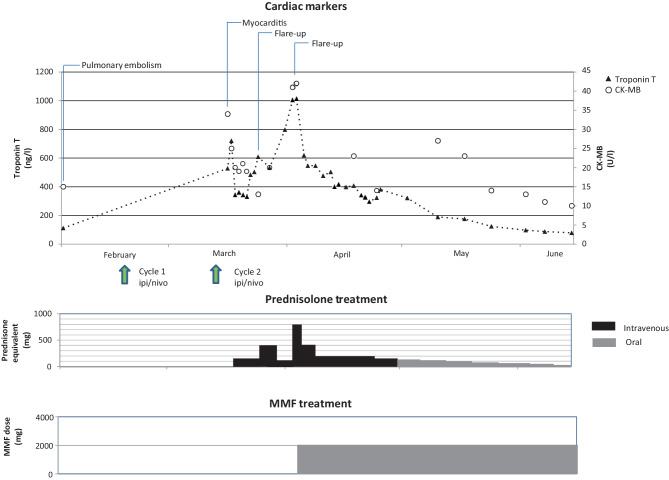
Time course of troponin T and creatine kinase myocardial band (*CK-MB*) levels during treatment with corticosteroids and mycophenolate mofetil (*MMF*) in case 1. *ipi* ipilimumab, *nivo* nivolumab

Within the next 24 h, the troponin T value decreased markedly (to 345 ng/l), and by day 5, there were no clinical signs of heart failure and all cardiac marker values were reduced (Fig. 2). Continuous telemetry showed a persistent sinus rhythm with a right bundle branch configuration. The temporary pacing wire was removed. After 7 days, the dose of prednisolone was tapered. This resulted in two consecutive rises in cardiac markers, without new symptoms or ECG changes. The significant rise in cardiac markers was thought to be related to a flare-up of the ICI-associated myocarditis due to prednisolone tapering.

After the first rise, the patient had been switched to methylprednisolone 500 mg/day intravenously, which was increased to 1000 mg/day after the second flare-up with the addition of mycophenolate mofetil (MMF) 1000 mg orally twice daily (BID). Tapering of the immunosuppressive therapy was thereafter guided by repeated troponin T measurements instead of tapering at predefined intervals of two to three days.

At day 21, cardiac magnetic resonance imaging (CMR) was performed, which showed delayed intramural enhancement of the basal and inferior wall, supporting the diagnosis of myocarditis. LVEF was 64% and there were no wall motion abnormalities. Global longitudinal strain (GLS) was not measured. During co-treatment with MMF, the methylprednisolone dose could be gradually decreased without significant rises in troponin T level. The patient was eventually discharged with prednisolone 160 mg/day orally, which was tapered to a dose of 50 mg/day over the course of eight weeks.

Unfortunately, follow-up imaging showed progression of renal cell carcinoma metastases. Twelve weeks after discharge, the patient was readmitted with pneumonia and abdominal sepsis. After deliberation with the patient, his family and the surgeons, it was eventually decided to stop all treatment and focus on symptom management. He succumbed to his disease approximately one week after discharge.

## Case 2

A 70-year-old patient with diabetes mellitus and chronic obstructive pulmonary disease was diagnosed with type 1 metastatic papillary renal cell carcinoma. He was included in a randomised phase II study and assigned to treatment with ipilimumab 1 mg/kg and nivolumab 3 mg/kg intravenously, every 3 weeks for four cycles (ClinicalTrials.gov NCT03075423) [6]. Per protocol, the troponin T level was measured before every cycle. At the visit for his second cycle, it was noted that the troponin T level had increased to 74 ng/l (normal < 14 ng/l), whereas the baseline level was normal (11 ng/l). In addition, he also showed elevated levels of CK 334 U/l (normal < 117 U/l), lactate dehydrogenase (LDH) 498 U/l (normal < 248 U/l) and aspartate aminotransferase (ASAT) 43 U/l (normal < 35 U/l).

He did not have any chest pain, but he had noticed dyspnoea on exertion. His ECG showed a first-degree AV block (PR interval 244 ms), which was pre-existent. ICI therapy was discontinued, and the patient was admitted to the CCU for observation, telemetry and echocardiography. The echocardiogram showed a LVEF of 55% with signs of diastolic dysfunction. GLS was not measured. Monitoring of the heart rhythm did not show any changes from the baseline ECG. Therefore, it was concluded that he had asymptomatic elevated troponin levels, possibly because of subclinical myocarditis.

The troponin T level gradually increased to 153 ng/l over the course of one week, and the patient was readmitted to the hospital. Cardiac positron emission tomography (PET)/computer tomography (CT) and CMR did not show any signs of myocarditis. Follow-up ECG, however, revealed a worsening of the first-degree AV block (PR interval 296 ms), and subsequent telemetry monitoring also indicated development of paroxysmal second-degree AV blocks. Given the continuing rise in cardiac markers, new ECG changes and absence of other explanations, ICI-associated myocarditis was diagnosed, and methylprednisolone was initiated at 500 mg/day intravenously. The cardiac-specific troponin I level was elevated (50 ng/l, normal < 18 ng/l), which further substantiated the diagnosis.

**Fig. 3:**
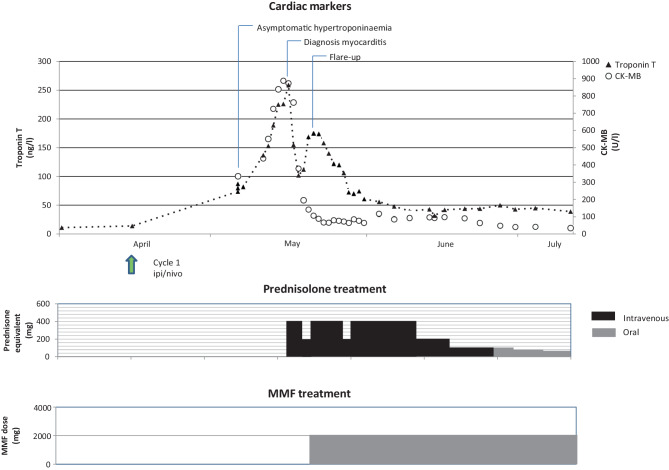
Time course of troponin T and creatine kinase myocardial band (*CK-MB*) levels during treatment with corticosteroids and mycophenolate mofetil (*MMF*) dosing in case 2. *ipi* ipilimumab, *nivo* nivolumab

After initiation of corticosteroid treatment, the troponin T level decreased within 24 h. In addition, prophylactic therapy for osteoporosis and for *Pneumocystis jirovecii* pneumonia was initiated. Similar to case 1, tapering of corticosteroids after three days caused a flare-up of troponin levels, but after addition of MMF (1000 mg BID), the troponin T level gradually decreased (Fig. 3).

During follow-up at the outpatient clinic, a follow-up CT scan showed stable disease with regard to the metastatic renal cell carcinoma. However, there were new cavitating pulmonary lesions. Over the course of six months, immunosuppressive therapy could be tapered and stopped. Unfortunately, progression of lymph node metastases was noted. Thereafter, second-line treatment with sunitinib, a tyrosine kinase inhibitor, was initiated.

## Review of the literature

As demonstrated by the described cases, ICI-associated myocarditis can present as a fulminant disease with serious arrhythmias but also as an asymptomatic troponin release. Both patients were eventually treated with high-dose corticosteroids and MMF. In this review, we focus on the diagnosis and treatment of ICI-associated myocarditis. We conducted a search in PubMed with the following search terms: ‘ICI’ OR ‘immunotherapy’ OR ‘Immune checkpoint’ AND ‘myocarditis’.

### Diagnosis of myocarditis

ICI-associated myocarditis is a rare complication, which occurs in 0.27–1.14% of patients who receive monotherapy. However, it is more frequent (up to 2%) in patients receiving combination therapy of anti–PD-(L)1 and anti–CTLA‑4 [7, 8]. Given the high mortality rate of symptomatic ICI-associated myocarditis (50%), early recognition is important [1, 7, 9]. This high mortality rate could be an overestimation, as mild cases may be underreported in current studies.

ICI-associated myocarditis develops early; in 62% of patients, it occurs after the first or second cycle of ICI therapy, with a median time to onset of 30 days (interquartile range 18–60) and 76% of cases occurring during the first six weeks of treatment [1, 10, 11]. Therefore, expert opinion–based diagnostic algorithms now tend to include screening for ICI-associated myocarditis during the first few treatment cycles using regular troponin T measurements [11, 12].

Although myocardial biopsy is the gold standard, it is not advised as the first diagnostic step due to its invasiveness and risk of complications [13–15]. Furthermore, the sensitivity of myocardial biopsy is limited by sampling error [16]. Alternatively, a combination of clinical symptoms, biochemistry and imaging can be used to diagnose ICI-associated myocarditis. Clinically suspected myocarditis involves a combination of the following: (1) a syndrome suggestive of possible myocarditis (e.g. acute chest pain, new or worsening dyspnoea or collapse); (2) abnormal diagnostic tests such as ECG changes, troponin elevation and/or abnormalities in cardiac imaging that are in accordance with myocarditis; and (3) exclusion of other causes (e.g. ischaemic heart disease, pulmonary embolism, pericarditis, myositis, valvular disorders and viral myocarditis) [12].

Regular measurement of troponin T is one of the easiest ways to screen for the development of myocarditis. The potential advantage of screening is early recognition of subclinical myocarditis and initiation of treatment prior to the development of severe cardiac symptoms. Limited retrospective data suggest that early treatment improves the outcome of patients with ICI-associated myocarditis, which argues for incorporation of repeated troponin T measurements in the daily clinic [8]. On the other hand, troponin rises can be nonspecific. No evidence-based cut-off points for troponin T in patients with possible myocarditis exist. Therefore, regular testing may lead to unnecessary discontinuation of immunotherapy and unnecessary start of immunosuppressive therapy [17]. Moreover, although ICI toxicity is associated with prolonged overall survival, it is still unknown if the combination of withholding ICI treatment and starting systemic immunosuppression abolishes the anti-tumour effect [18]. This makes it important to prospectively evaluate the results and consequences of repeated troponin T testing.

In patients with an asymptomatic but significant rise of troponin T, it is currently advised to temporarily hold the ICI therapy to perform serial measurements of CK, CK-MB and troponin T, perform an ECG and consult a cardiologist. If all markers stabilise or normalise within two weeks, it is assumed that ICI therapy can be safely resumed. However, if the troponin T level continues to rise or ECG changes develop, myocarditis should be suspected, and immunosuppressive treatment is recommended. In cases with uncertain diagnosis, troponin I, repeated echocardiography, and CMR or [^18^F]-fluoro-2-deoxy-D-glucose (FDG) PET may be used to further support the diagnosis. The need for additional diagnostic tests should, in these circumstances, be carefully weighed against the risk of delaying immunosuppressive therapy for myocarditis. This is a real problem, as troponin levels tend to remain elevated for weeks rather than days. Confounders such as age, sex and especially renal function may further complicate interpretation of serum troponin levels.

The most common concurrent immune-related adverse events in patients with ICI-associated myocarditis are myositis (25%) and myasthenia gravis (11%) [11]. Troponin I can be of additional value in distinguishing ICI-associated myositis from ICI-associated myocarditis. Measurement of troponin I is therefore recommended if the troponin T level is elevated in the absence of cardiac symptoms or in the presence of either other elevated muscle enzymes or clinical features of active skeletal muscle disease [19]. Troponin I is considered to be exclusive to myocardial tissue. In contrast, troponin T is also released by healthy and regenerating adult skeletal muscle tissue, and its level is elevated in patients with idiopathic or ICI-associated inflammatory myopathies [19].

An echocardiogram may show wall motion abnormalities and a reduced LVEF. However, a normal LVEF is found in 36–51% of cases, and a normal LVEF does not rule out ICI-associated myocarditis [8, 10]. Therefore, GLS has gained recent attention in the detection of cardiotoxicity [20]. A lower GLS is strongly associated with major adverse cardiac events in patients with ICI-associated myocarditis with either a preserved or reduced LVEF [21].

CMR is superior to echocardiography, as it provides better tissue characterisation both with and without gadolinium contrast. Features of myocarditis on CMR include oedema, necrosis and fibrosis, as defined by the Lake Louise criteria [16]. The diagnostic accuracy of CMR has been improved by combining oedema-sensitive cardiovascular imaging (T_2_-weighted images) with at least one additional T_1_-based tissue characterisation technique [16]. The absence of late gadolinium enhancement or the absence of increased T_2_-weighted signal on CMR does not exclude ICI-associated myocarditis, as late gadolinium enhancement is present in < 50% of patients with ICI-associated myocarditis [22]. It should be noted that T_1_- and T_2_-based CMR is not readily available in all centres and sequences used are not standardised, making interpretation difficult [16, 23].

Cardiovascular adverse events caused by ICI therapy can be defined according to the Common Terminology Criteria for Adverse Events or the American Society of Clinical Oncology (ASCO) clinical practise guidelines, as shown in Tab. 1; [5, 24]. The American Heart Association (AHA) has recently published a scientific statement on the recognition and initial management of ICI-associated myocarditis [25].

**Table 1:** Cardiovascular toxicity classification according to the ASCO and CTCAE guidelines

Guideline (year)	Grade 1	Grade 2	Grade 3	Grade 4	Grade 5
ASCO (2018)	Abnormal cardiac biomarker testing, including abnormal ECG	Abnormal screening tests with mild symptoms	Moderately abnormal testing or symptoms with mild activity	Moderate to severe decompensation; life-threatening conditions; IV medication or intervention required	–
CTCAE version 5.0 (2017)	–	Symptoms with moderate activity or exertion	Severe with symptoms at rest or with minimal activity or exertion; intervention indicated; new onset of symptoms	Life-threatening consequences; urgent intervention indicated (e.g. continuous IV therapy or mechanical hemodynamic support)	Death

*ASCO* American Society of Oncology, *CTCAE* Common Terminology Criteria for Adverse Events, *ECG* electrocardiogram, *IV* intravenous

### Treatment of ICI-associated myocarditis

The treatment strategy for ICI-associated myocarditis consists of three parts: (1) interrupting ICI treatment to prevent further toxicity; (2) immunosuppression with prednisolone or other immunosuppressive agents to inhibit the inflammatory process; and (3) patient monitoring to assess the development of any cardiac complications. Here, we focus on the immunosuppressive strategy.

The AHA, the European Society for Medical Oncology and the ASCO guidelines advise to start with intravenous prednisolone or methylprednisolone at 1–2 mg/kg per day in patients with mild to moderate symptoms (toxicity grades 2 and 3; see Tab. 1; [5, 25, 26]). Patients with more severe disease (toxicity grades 3 and 4) and those who fail to respond to initial corticosteroid dosing within 3–5 days should be switched to methylprednisolone (1000 mg daily).

Recently, the results were published from a retrospective observational multicentre study, which included a small subset of 35 patients who developed ICI-associated myocarditis with or without major cardiac events [8]. Patients who developed major adverse cardiac events (defined as cardiovascular death, cardiac arrest, cardiogenic shock, and haemodynamic instability due to significant heart block; *n* = 16) received a lower initial corticosteroid dose and had a longer time interval from admission to corticosteroid administration than those who did not develop major adverse cardiac events (*n* = 19) [8]. However, given the retrospective nature, these results should be interpreted with caution. Nonetheless, higher corticosteroid dosage is associated with a higher probability of left ventricular function recovery [27]. Therefore, our local approach is prompt treatment with methylprednisolone 1000 mg intravenously daily in all patients regardless of grade of myocarditis, which may be tapered after three days in mild cases [28].

If first-line immunosuppression with intravenous methylprednisolone is unsuccessful—defined as insufficient control (e.g. further increase of troponins, new symptoms or ECG changes) after 48 h—second-line immunosuppression with MMF, tacrolimus, anti-thymocyte globulin, intravenous immunoglobulins or plasmapheresis should be considered [10, 28]. However, these treatment recommendations are based on anecdotal evidence [5]. Some authors advocate infliximab. However, treatment with this anti–tumour necrosis factor‑α antibody has been associated with heart failure, and high doses are contraindicated in patients with moderate-severe heart failure. Given the availability of various other second-line agents, the use of infliximab should therefore be discouraged. In our protocol, MMF (1000 mg BID) is added as second-line treatment, because it is supported by most clinical evidence. Preclinical data show a rapid anti-inflammatory effect within 24 h [29].

If myocarditis is refractory to treatment with second-line agents, the diagnosis should be reviewed, and other treatment options may be considered. As there are pathophysiological and histological similarities between ICI-associated myocarditis and cardiac transplant rejection, anti–transplant rejection medication, including anti-thymocyte globulin, has been successfully used [30]. In addition, recent reports have shown successful (off-label) treatment with abatacept (CTLA‑4 agonist) or alemtuzumab in patients with corticosteroid-resistant ICI-associated myocarditis [31, 32]. Alemtuzumab is a monoclonal antibody that binds to CD52. It leads to complement-mediated destruction of peripheral immune cells [31].

Future studies are needed to provide better guidance as to the most effective treatment of ICI-associated myocarditis. Furthermore, reporting and registration of major adverse events of ICI therapy remain essential. Rare or late complications can remain undetected in phase III studies. Therefore, databases such as the World Health Organization’s VigiBase are of great importance [33]. Individual case safety reports are registered in this global database, thereby ensuring that early signs of previously unknown medicine-related safety problems or adverse events are identified as rapidly as possible.

## Conclusion

ICI-associated myocarditis is a rare but serious complication that should trigger further diagnostic steps and treatment. In this review, we have described the challenges in recognising, diagnosing and treating ICI-associated myocarditis, including mild troponin elevations in case of subclinical myocarditis. If the diagnosis of ICI-associated myocarditis made, ICI therapy should be interrupted, and prompt treatment with methylprednisolone should be initiated. At this time, there are no clear data on the preferred second-line treatment if methylprednisolone is unsuccessful. Therefore, future studies are needed, while registration of complications in databases such as VigiBase remains of great importance.
